# A Mapping Review on the Uptake of the COVID-19 Vaccine among Adults in Africa Using the 5As Vaccine Taxonomy

**DOI:** 10.4269/ajtmh.21-0515

**Published:** 2022-05-09

**Authors:** Michael E. Kalu, Oluwagbemiga Oyinlola, Michael C. Ibekaku, Israel I. Adandom, Anthony O. Iwuagwu, Chigozie J. Ezulike, Ernest C. Nwachukwu, Ekezie Uduonu

**Affiliations:** ^1^ Emerging Researchers and Professionals in Ageing–African Network, Hamilton, Ontario, Canada;; ^2^ School of Rehabilitation Science, McMaster University, Hamilton, Ontario, Canada;; ^3^ Medical Social Services Department, University College Hospital Ibadan, Ibadan, Nigeria;; ^4^ University of Benin Teaching Hospital, Benin, Nigeria;; ^5^ Cedarcrest Hospitals, FCT-Abuja, Nigeria;; ^6^ Department of Social Work, University of Nigeria, Nsukka, Nigeria;; ^7^ Department of Social and Behavioral Sciences, City University of Hong Kong, Kowloon, Hong Kong;; ^8^ Physiotherapy Department, Enugu State University of Science and Technology Teaching Hospital, Parklane-Enugu;; ^9^ Department of Medical Rehabilitation, Faculty of Health Sciences and Technology, College of Medicine, University of Nigeria, Enugu Campus

## Abstract

Uptake of a vaccine is complete if individuals are aware of the associated risks of the vaccine, accept the vaccine, and respond positively to the nudges (activation) to increase the uptake, and respond when the vaccine is made accessible and affordable. We mapped systematically the existing literature concerning the 5As—acceptability, accessibility, affordability, awareness, and activation—of COVID-19 vaccination among adults and, specifically, older adults (55 years and older) in Africa. We searched multiple databases from 2020 to December 2021. Using predefined inclusion and exclusion criteria, two reviewers screened citations, conducted title and abstract screening, and extracted data independently. We included 68 articles conducted in 33 African countries, primarily cross-sectional studies (*n* = 49, 72%). None of the articles focused on older adults only, but 22 articles (32%) included at least one older adult (55 years and older) in their sample size. Acceptance (*n* = 58, 85%) was the most commonly researched aspect of vaccine uptake, followed by accessibility (*n* = 17, 25%), awareness (*n* = 13, 19%), and affordability (*n* = 5, 7.0%). We found only one report on activation. Factors affecting acceptance of the COVID-19 vaccine in Africa were grouped into sociodemographic factors; knowledge-, attitude-, and belief-related factors; a COVID-19 vaccine efficacy and safety concern factor; and trust in government and public health authorities. The governments of African nations should focus on strategies to influence the modifiable factors identified in this review. More studies are needed to evaluate the impact of nudges (activation) to improve COVID-19 vaccine uptake in African nations.

## INTRODUCTION

Deaths from COVID-19 infection are likely to reduce if we achieve herd immunity—when 60% to 80% of the world population has received the two doses of the COVID-19 vaccine.^1^ Vaccine hesitancy, defined as a delay in acceptance or refusal of the vaccine even if available, is a growing health issue globally that presents an obstacle to achieving substantial uptake compliance of any new vaccine.^2^ The determinants of vaccine hesitancy include acceptability, accessibility, affordability, awareness, and activation^3^; these are the myriad possible root causes of vaccine uptake.^3^

Vaccine hesitancy results from a complex decision-making process influenced by various environmental, agent, and personal factors.^4^ Environmental factors include, but are not limited to, public health policies related to the design of vaccination programs, and messages spread by the media; agent factors include the perception of vaccine safety, effectiveness, and disease risk.^4^ Personal factors include knowledge, previous historical experience, socioeconomic status, and religious belief.^4^ These factors have been explored in studies on COVID-19 vaccine uptake.^5^^,^^6^ The study by Lin et al.^6^ included 126 studies in a rapid systematic review and reported 1) declining COVID-19 vaccine acceptance (from > 70% in March to < 50% in October) with demographic, socioeconomic, and partisan divides; and 2) perceived risk, concerns over vaccine safety and effectiveness. Doctor recommendation and inoculation history were common factors that influenced vaccine acceptance. Similarly, Sallam^5^ included 31 peer-reviewed articles from 33 countries and reported a low rate of COVID-19 vaccine acceptance in the Middle East, Russia, Africa, and several European countries. Two reviews have been conducted to explore the extent and determinants of COVID-19 vaccine hesitancy in South Africa^7^ and to determine the acceptance rate of the COVID-19 vaccine in Africa.^8^ Although the findings from the reviews have provided information to guide interventional measures aimed at increasing acceptance, other determinants of vaccine hesitancy, including access, affordability, awareness, and activation, are largely ignored. Therefore, a more comprehensive review is needed to highlight how other determinants have reduced vaccine hesitancy across African nations. Older adults are at a greater risk of contracting and dying from the COVID-19 virus; therefore, our review is situated in the context of the older population in Africa.

Gerontological studies conducted in Africa have chosen ages 50, 55, 60, or 65 as a lower limit to be considered old.^9^ However, in this article, we define older adults as 55 years and older, because this value aligns with the Africa Centers for Disease Control and Prevention reports.^10^ Older adults are at a greater risk of contracting and dying from COVID-19 infection. Therefore, older adults and individuals living with multiple chronic conditions should be the first category of individuals to receive the COVID-19 vaccine. Although this is true in developed regions of the world, this is not the case in some African countries. For instance, according to Africa Centers for Disease Control and Prevention, countries such as Nigeria, South Africa, Namibia, and Zimbabwe did not list older adults (50 years and older) as their priority group.^10^ Reasons for not listing adults 50 years and older as a priority group could include a lack of implementation of health-care access policies targeted towards older adults or loose policies that cannot guide detailed program health-care access.^11^^,^^12^ These reasons are further influenced by the lack of conviction that old-age-related health should be a priority in the context of diverse, pressing public health needs for women and children that currently account for the highest proportion of the population for most African nations.^13^ Not placing older adults as a priority group in some African countries adds even more challenges to the already existing issues regarding vaccination, such as accessibility and acceptance. The primary objective of this review was to map the existing literature concerning the 5As—acceptability, accessibility, affordability, awareness, and activation of the COVID-19 vaccination—among adults 55 years and older in Africa. We assumed that studies exploring these concepts among older adults in Africa might not be extensive; therefore, we expanded our search to include adults 18 years and older. Thus, the secondary aim was to map the existing literature across the 5As for adults 18 years and older in Africa. First we describe the current state of the COVID-19 vaccination in Africa. Second, based on our reviewed literature, we explain the current COVID-19 vaccination uptake in Africa using Thomson’s 5As taxonomy.^3^ Gaps in the literature to ensure the uptake of COVID-19 vaccination using the 5A taxonomy are provided.

## COVID-19 VACCINATION IN AFRICA

**Figure 1.**
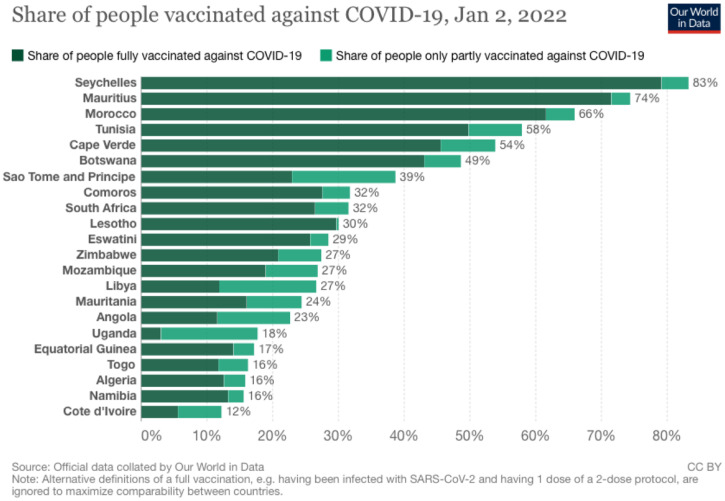
Share of people vaccinated against COVID-19, as of January 2, 2022. Source: https://ourworldindata.org/covid-vaccinations. This figure appears in color at www.ajtmh.org.

As of January 2, 2022, 56.90% of the vaccine supplied has been administered, and 13.65%, 9.11%, and 0.31% of the African population have received one vaccine, two vaccines, and the booster dose of the COVID-19 vaccine, respectively.^10^ According to Africa Centers for Disease Control and Prevention vaccine dashboard,^10^ all African countries have received the COVID-19 vaccine and commenced vaccination except for Eritrea. Twenty countries have fully vaccinated at least 10% of their population (Figure 1); other African countries not shown in Figure 1 have less than 10% of their population fully or partially vaccinated.^14^ Several countries received vaccines from COVID-19 Vaccine Global Access (COVAX) (50 countries), African Union's COVID-19 Africa Vaccine Acquisition Task Team (AVATT) (38 countries), and both (50 countries). COVID-19 vaccine types currently administered in African countries are AstraZeneca, Sinopharm, Sputnik V, BioNTech, Sinovac, Moderna, and Covaxin.^10^

**Figure 2.**
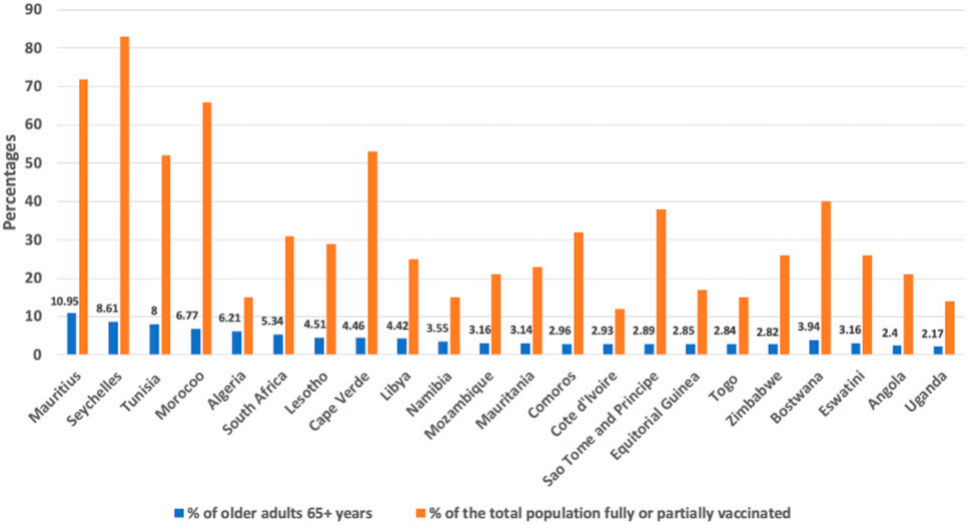
Percentage of older adults alongside percentage of the total population fully or partially vaccinated as of January 2, 2022, Source: Our World in Data, 2022. *Coronavirus (COVID-19) Vaccinations*. Source: https://ourworldindata.org/covid-vaccinations. This figure appears in color at www.ajtmh.org.

Across African countries, the priority groups to receive the COVID-19 vaccine include health-care workers, people with chronic conditions, people 50 years and older, front-line/essential workers, and leaders/prisoners.^10^ Although health-care workers were consistently a priority group to receive the COVID-19 vaccine, people 50 years and older were not among the priority group in some African countries, including South Africa, Zimbabwe, Nigeria, Botswana, Mozambique, Angola, and Cameroon.^10^ Not including the older adult population is a concern for several reasons. First, older adults inherently have compromised immunity as a result of accumulated health disadvantages across their life course. Second, most older adults in Africa reside in rural areas with little or no access to health-care facilities and, by extension, no access to the COVID-19 vaccine. The percentage of adults 55 years and older across the 22 countries with at least 10% of their population fully or partially vaccinated is presented in Figure 2.

Based on the definitions of Thomson et al.,^3^ accessibility is a country’s/individual’s ability to obtain a vaccine within a reasonable reach, whereas affordability is defined as economic accessibility (i.e., a country/individual can pay for vaccines with minimal financial and non-financial cost [e.g., time]). Awareness is the degree to which individuals know the need for and availability of recommended vaccines, and their associated risks and benefits.^3^ Although acceptance is the degree to which individuals recognize or agree to take and/or question a vaccine, activation is the extent to which individuals are nudged toward vaccine uptake.^3^

Among the 5As of acceptability, accessibility, affordability, awareness, and activation, activation is rarely discussed among scholars or even in African countries’ media outlets. Although acceptance, access, and affordability are often discussed, culturally motivated awareness programs that are age friendly are rare. Therefore, we reviewed the literature systematically to describe how many studies have explored each component of the 5As with regard to COVID-19 vaccination among the older adult population in Africa.

## METHODS

We followed Grant and Booth’s^15^ description of mapping review to categorize existing literature from which to commission further reviews and/or primary research by identifying gaps in the literature. We identified literature of aging with regarding to COVID-19 vaccine-related issues in Africa and mapped the existing literature across the 5As: acceptability, accessibility, affordability, awareness, and activation.

### Search.

We did a preliminary search in the Cumulative Index to Nursing and Allied Health Literature (CINAHL) including aging terms such as aging, older adults, older people, and other search terms described later. This search yielded no articles. We repeated the exact search in PubMed, using the COVID-19 vaccine filter “treatment and prevention,” and 230 articles were identified. None of these articles focused on vaccine uptake among older adults in Africa. We chose CINAHL and PubMed for our preliminary search because of their extensive index citations and a recommendation of them as being comprehensive databases for reviews.^16^ We repeated our search removing the aging terms and examined the databases PubMed and Ovid (EMBASE, CINAHL, and PsychINFO) from 2020 to December 2021 (see Table 1 for our search strategy).

**Table 1:** Key terms adapted for each database

Major term	Term
Vaccine uptake	Accessibility *or* obtainability *or* gain access *or* availability *or* location *or* convenience *or* affordability *or* awareness *or* acceptance *or* activation *or* cost *or* economical *or* vaccine uptake
COVID-19	Covid-19 *or* coronvarius-19 *or* SARS-COV-2
African country	Egypt *or* Morocco *or* Tunisia *or* Algeria *or* Libya *or* Somalia *or* Mali *or* Angola *or* Ethiopia *or* Nigeria *or* Niger *or* Benin *or* Gabon *or* Botswana *or* The Gambia, *or* Rwanda *or* Burkina Faso *or* Ghana *or* Sao Tome and Principe *or* Burundi *or* Guinea *or* Senegal *or* Cabo Verde *or* Guinea-Bissau *or* Seychelles *or* Cameroon *or* Kenya *or* Sierra Leone *or* Central African Republic *or* Lesotho *or* Somalia *or* Chad *or* Liberia *or* South Africa *or* Comoros *or* Madagascar *or* South Sudan *or* Congo, Dem. Rep. *or* Malawi *or* Sudan *or* Congo, Rep *or* Mali *or* Tanzania *or* Cote d’Ivoire *or* Mauritania *or* Togo Equatorial Guinea *or* Mauritius *or* Uganda *or* Eritrea *or* Mozambique *or* Zambia *or* Eswatini *or* Namibia *or* Zimbabwe

### Study selection.

All citations from each database were exported into Rayyan Qatar Computing Research Institute (QCRI).^17^ After removing duplicates, studies were conducted in two stages: title/abstract and full-text screening. Two raters performed a pilot test independently, which consisted of a title/abstract and full-text screening of the first 50 articles using predefined inclusion and exclusion criteria to determine interrater reliability. Light’s kappa of the raters for both title/abstract and full-text screening were 0.88 and 0.92. These kappa values indicate an almost perfect strength of agreement^18^; thus, retrieved articles were divided among the two raters. Questions, concerns, and disagreements at any stage were discussed during research meetings.

We included articles focused on the concepts related to COVID-19 vaccine uptake as guided by the 5As by Thomas et al.^3^ There was no restriction on the type of study design; quantitative, qualitative, and mixed-method studies were included. Because of the limited number of articles in this subject area, we included opinions and editorials that discussed ideas regarding COVID-19 vaccine uptake among older adults in Africa. We searched the included articles, the *African Journals Online *portal,^19^ WHO websites,^20^ the *African Union Center for Disease Control and Prevention*,^10^ and country-specific centers for disease control, such as the Nigeria Center for Disease Control^21^ and the South Africa Department of Health.^22^

Articles were excluded if the study population or discussion did not focus on Africans and were not published in English or French.

### Data extraction.

We adopted a standardized data extraction sheet used in a previous scoping review^23^ to extract data from the included studies. We “pilot-tested” the data extraction process in a research meeting. Two reviewers extracted the authors’ names; the country in which the study was conducted; study aims, research question(s), and hypotheses; study setting; type of study (qualitative, quantitative, mixed method, editorials, and gray literature); study design; sampling method; participant characteristics (e.g., number of participants, mean age of the participants); main study findings; and recommendations (policy, practice, and research).

### Collating, summarizing, and reporting the results.

We used the Preferred Reporting Items for Systematic Reviews and Meta-Analyses (PRISMA) flowchart to describe the process of data selection. The best-fit framework synthesis guided the grouping of the included studies.^24^ This approach allowed us to map the studies across the 5As: acceptability, accessibility, affordability, awareness, and activation. Two authors read the included studies and mapped them independently across the 5As, noting those articles that did not apply. Both authors met and discussed their mapping; discrepancies were discussed in a research team meeting.

## RESULT

**Figure 3.**
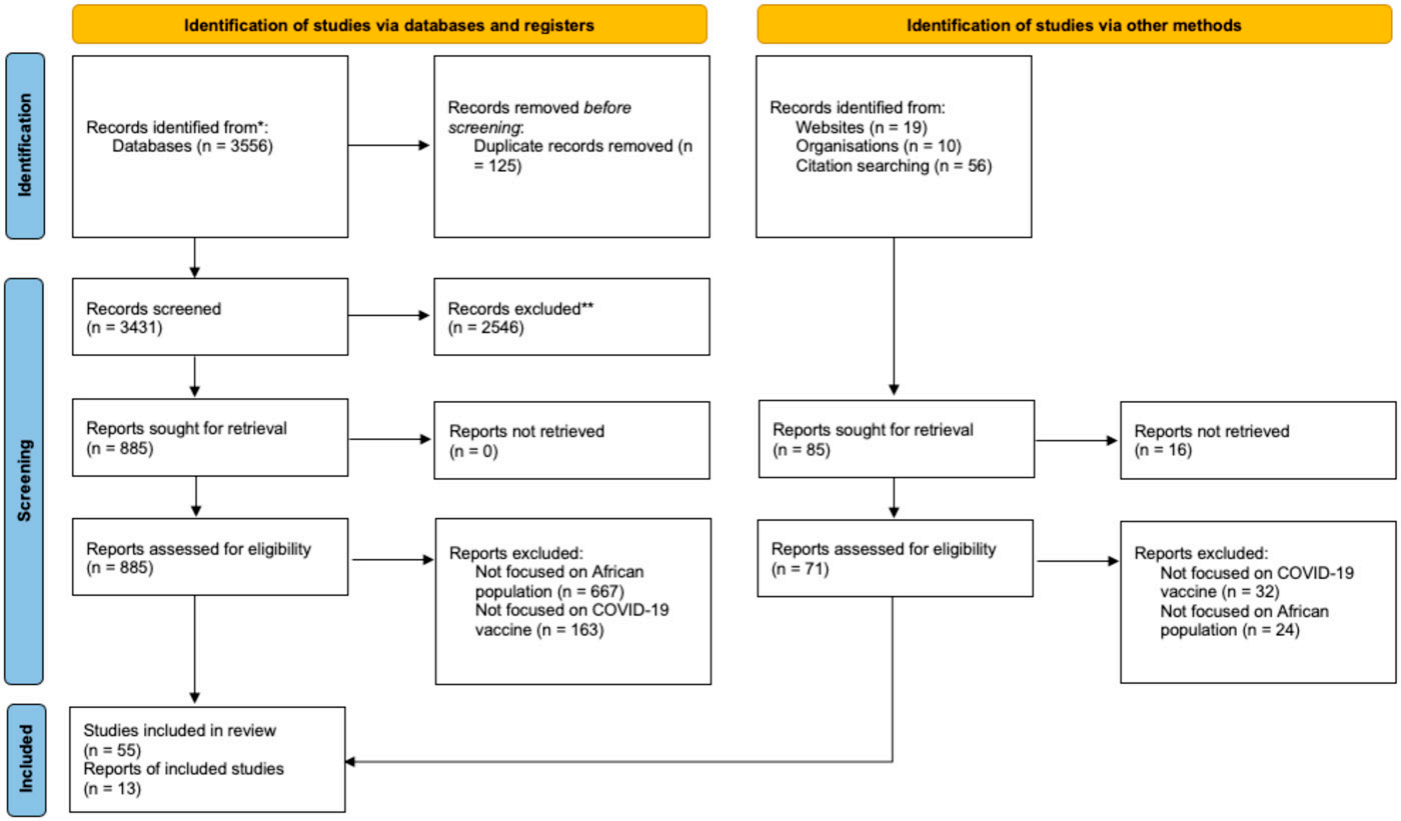
PRISMA chart indicating study selection process. Source: Page MJ et al., 2021. The PRISMA 2020 Statement: an updated guideline for reporting systematic reviews. *Systematic Reviews 10(89):* 1–11. This figure appears in color at www.ajtmh.org.

We retrieved 3,556 studies from our database search. After removing duplicates, 3,431 citations underwent abstract and title screening, which resulted in the exclusion of 2,546 studies. The remaining 885 studies underwent full-text screening; we included 53 studies. An additional 15 studies from hand-searching included studies, African journals, and websites, resulting in 68 articles (Figure 3).

### Description of included studies.

**Figure 4.**
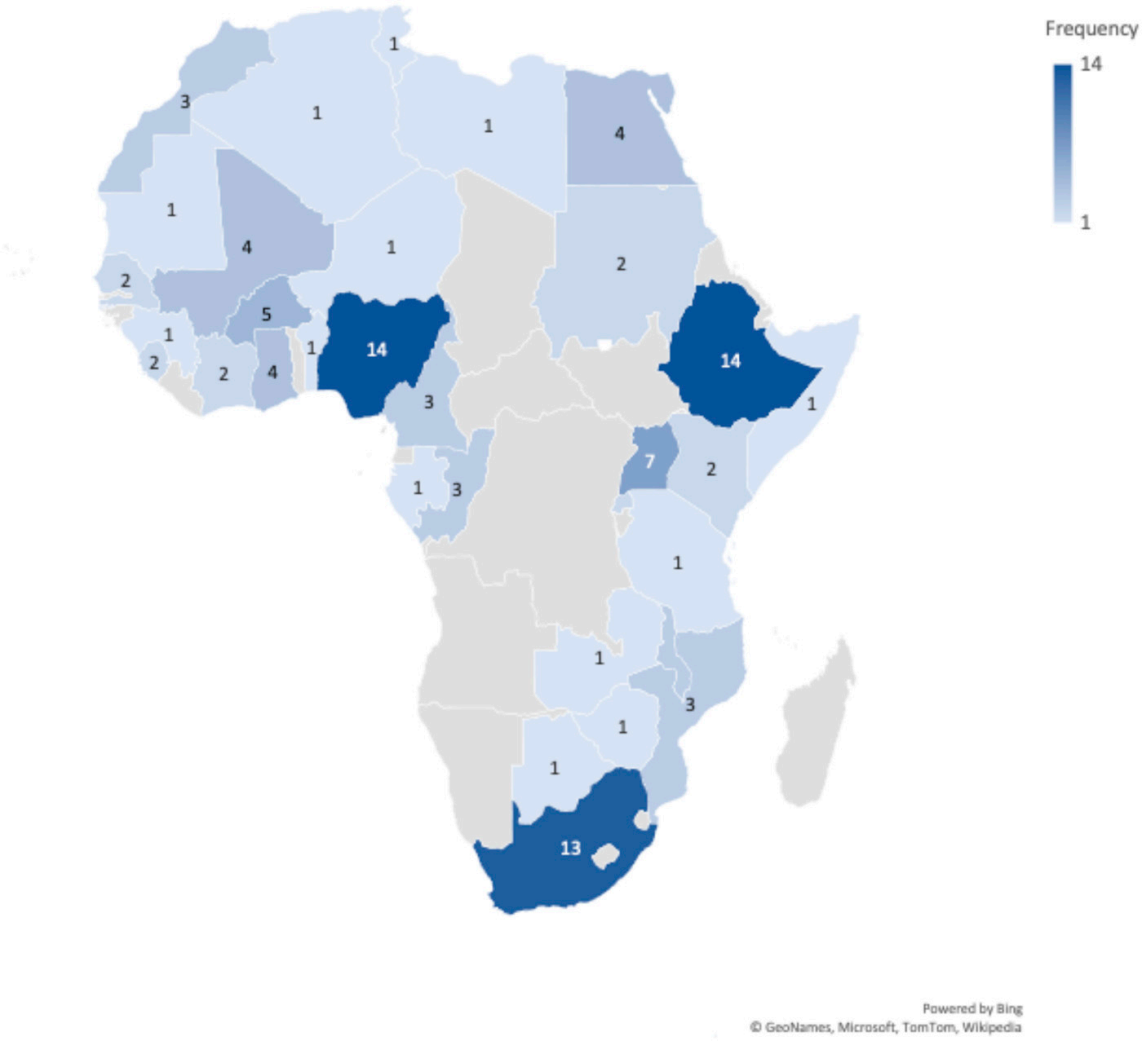
Countries and number of articles that have discussed at least one of the 5As—acceptability, accessibility, affordability, awareness, and activation—of the COVID-19 vaccine in Africa. This figure appears in color at www.ajtmh.org.

The 68 articles included in this review are presented in Supplemental Table S1. Included articles/reports were from 33 African countries (Figure 4): from one single African country (*n* = 49, 72%), multiple African countries (*n* = 11, 16%),^25^^–^^36^ or no specific African country (*n* = 8, 12%).^37^^–^^44^ Almost three quarters of the included articles were cross-sectional studies (*n* = 50, 74%), whereas the remaining were mixed method (*n* = 4, 6%) and opinion papers or commentaries (*n* = 14, 20%). None of the articles focused on older adults only, but 22 (32%) studies included adults 55 years and older in their sample size. Among the cross-sectional and mixed-methods studies (*n* = 54, 79%), most surveyed the general population (*n* = 34, 63%), followed by health-care workers (*n* = 13, 24%) and specific populations (*n* = 7, 12%), such as people living with HIV,^45^ pregnant women,^46^^,^^47^ adult caregivers,^48^ and medical students.^49^^–^^51^

### Mapping the existing literature across Thomas’s 5As.

Close to three quarters of the included reports (*n* = 43, 63%) focused only on acceptance (*n* = 49) or accessibility (*n* = 4) of the COVID-19 vaccine. The remaining 25 reports focused on at least two of the 5As.

#### Acceptance.

Fifty-eight articles reported on acceptance, with acceptance percentages of COVID-19 vaccine ranging from 15.4%^52^ to 90%.^31^ Among the 58 articles, nine studies reported ≥ 80%^26^^,^^27^^,^^31^^,^^32^^,^^36^^,^^53^^–^^56^ of their participants would accept/take the COVID-19 vaccine when available, 27 studies reported between 50% and 79%,^28^^–^^30^^,^^34^^–^^36^^,^^46^^,^^47^^,^^51^^,^^57^^–^^73^ and 15 studies reported < 50%.^36^^,^^45^^,^^49^^,^^50^^,^^52^^,^^74^^–^^82^

Of 58 studies, 31 reported factors that influence the desire to be vaccinated and were grouped as 1) sociodemographic factors, including gender (male^45^^,^^49^^,^^50^^,^^60^^,^^64^^,^^67^^,^^69^^,^^70^^,^^80^^–^^83^ or female^75^), age (old age [55 years and older],^75^^,^^83^ middle age [34–41 years],^46^^,^^69^ and younger age [18–20 years]^27^^,^^34^^,^^64^^,^^82^), marital status,^49^^,^^64^^,^^75^ higher educational status,^46^^,^^64^^,^^69^^,^^70^^,^^75^^,^^81^^,^^82^ occupation,^59^^,^^75^ higher income,^27^^,^^61^ and religion and rural residence^64^^,^^75^; 2) knowledge-, attitude-, and belief-related factors, including COVID-19 knowledge of prevention practices and the vaccine,^27^^,^^45^^,^^46^^,^^50^^,^^61^ lack of adequate information (related to conspiracy theories),^58^^,^^66^^,^^79^^,^^81^ and how attitude/belief influences vaccine willingness to take the COVID-19 vaccine^25^^,^^27^^,^^53^^,^^57^^,^^78^^,^^79^; 3) efficacy and safety concerns, including concern over safety/side effects of the vaccine^26^^,^^29^^,^^55^^,^^58^^,^^65^^,^^79^^,^^84^ or doubts about vaccine effectiveness^27^^,^^55^^,^^58^^,^^67^^,^^74^^,^^79^; and 4) others, including the desire to protect oneself or others,^27^^,^^66^^,^^81^ a perception of being at low risk,^69^^,^^80^ previous history of vaccination as a child,^81^ lack of direct patient contact,^82^ working in rural areas,^69^ trust in government and public health authorities, and willingness to pay for and travel to get a COVID-19 vaccine.^83^

#### Accessibility.

Sixteen articles that discussed access to the COVID-19 vaccine in Africa were based on 1) location, which was described as the distance to and from COVID-19 vaccination centers^30^^,^^35^^,^^37^^,^^38^^,^^42^^,^^85^; 2) factors that could promote access to the COVID-19 vaccine, including developing African-focused coordination^44^^,^^86^ or international cooperation^41^^,^^87^^,^^88^ for the distribution of the COVID-19 vaccine, and vaccine confidence^89^^,^^90^; 3) government-related factors, such as refusal to provide the COVID-19 vaccine despite international aid,^91^ inequitable distribution of the COVID-19 vaccine,^29^^,^^30^^,^^42^ and general accessibility issues.^43^^,^^73^^,^^90^

#### Affordability.

Five reports (four opinion papers^37^^,^^44^^,^^85^^,^^86^ and one cross-sectional study^73^) discussed the affordability of the COVID-19 vaccine in Africa. Although Afolabi and Ilesanmi^85^ highlighted the COVAX program as a viable strategy for low-income countries in Africa that may not have the funds to purchase the COVID-19 vaccine, Acharya et al.^37^ elucidated that low socioeconomic status can affect vaccine purchase, resulting in an inequitable distribution of the COVID-19 vaccine. Choi^86^ provided unique information that the African Export-Import Bank offers loans to individual African Union member states to finance the immunization program. Nkengasong et al.^44^ stated that an African-focused coordinated approach is needed to purchase the COVID-19 vaccine. One study surveyed Nigerians, asking if they would purchase the COVID-19 vaccine if need be; 18% of participants reported they would be willing to pay for the COVID-19 vaccine.^73^

#### Awareness.

Thirteen articles reported on awareness of the COVID-19 vaccine, of which only four assess participant knowledge. Participant knowledge of the COVID-19 vaccine varies: 98%^62^ and 67%^73^ in a Nigerian population, and 74%^57^ and 53%^56^ in an Ethiopian population. Other authors focused on describing how information concerning COVID-19 vaccine efficacy and side effects should be made known to the populace^25^^,^^38^^,^^41^^,^^49^^,^^71^^,^^75^^,^^85^ or how knowledge of the COVID-19 vaccine could influence willingness to accept the COVID-19 vaccine.^28^^,^^78^

#### Activation.

One study noted that participants were nudged toward using protective measures against the COVID-19 virus rather than getting the COVID-19 vaccine.^79^

## DISCUSSION

We initially set out to map literature concerning the 5As—acceptability, accessibility, affordability, awareness, and activation of the COVID-19 vaccine—among older adults in the African region. No article explored any of the 5As for older adults alone; however, 38% of the studies included older adults (55 years and older) in their sample size. Older adults are at a greater risk of contracting and dying from the COVID-19 virus; therefore, most governments of African nations, especially those with an increasing number of older adults (e.g., Nigeria and South Africa), should revisit their priority list. Furthermore, studies exploring factors and determinants of vaccine hesitancy among older adults in Africa are warranted. Interestingly, the 5C scale of the Betsch et al.^92^ and the Vaccination Attitudes Examination scale^93^ have shown good internal reliability, and convergent, discriminant, and concurrent validity among the older adult population.

We included 68 articles when we extended our review to reports that explored the 5As among adults 18 years and older. Consistent with the review by Thomas et al.,^3^ acceptance was the most common research aspect of COVID-19 vaccine uptake among adults in Africa. The ease of accessing acceptance could explain why acceptance is studied frequently among researchers. For instance, most studies accessed acceptance with a single question: Are you willing to take the COVID-19 vaccine when available? Regardless, the success of the COVID-19 vaccination often depends on peoples’ willingness to receive the vaccination. Consistent with previous reviews,^94^^,^^95^ we grouped factors that influence the acceptability of the COVID-19 vaccine into sociodemographic, knowledge related, efficacy, and safety concerns. Factors that could promote access to the COVID-19 vaccine in Africa include international cooperation, vaccine confidence, and government-related factors. This grouping and its specific factors highlight the need for policymakers to focus on modifiable factors that could promote the acceptability and accessibility of the COVID-19 vaccine to promote herd immunity.^95^ To predict achieving herd immunity of at least 75%,^96^ longitudinal studies should explore whether the baseline prevalence acceptance rate is a predictor of completing the required COVID-19 vaccine doses, including the booster dose.

Accessibility and affordability are closely related concepts in vaccine uptake. Accessibility, in the context of this view, focused on reasonable reach, defined by distance to and from vaccination centers, whereas affordability refers to economic accessibility. At the individual level, affordability is currently not an issue in the uptake of the COVID-19 vaccine in Africa because individuals are not required to pay for the vaccine. However, different governments in African countries purchase the COVID-19 vaccine for their citizens. Interestingly, the COVAX initiative, co-led by the WHO, the Coalition for Epidemic Preparedness Innovations, and Gavi, the Vaccine Alliance, promises to offer equitable access to vaccines to low- and middle-income countries through a highly subsidized price.^44^ Currently, we are not aware of where individuals are being asked to pay for the COVID-19 vaccine in Africa. Although the direct cost of the COVID-19 vaccine is being borne by the governments of African countries, Ilesanmi et al.^73^ surveyed 440 participants in Nigeria to describe their willingness to pay for the COVID-19 vaccine. They reported that 81 respondents (18.40%) were willing to pay for the prospective COVID-19 vaccine because of their need to stay healthy. Of these individuals willing to pay, 45 (55.6%) were willing to pay at least 5,000 NGN ($US13.16). This study provided a glimpse into people’s willingness to pay for the vaccine. In some African countries, residents/citizens do not have access to the health services they need, when and where they need them, without incurring financial hardship. Therefore, residents/citizens pay for health-care services. Although the COVID-19 vaccine has been shown to induce a robust immune response toward the virus, its duration is still inconclusive.^97^ However, studies have reported that waning of neutralizing antibody levels occurs ∼6 months post-vaccination.^98^^,^^99^ With so many unknowns about the virus and its continuous mutations, it is plausible that several booster shots will be required. Therefore, the African nations’ governments may have limited funding to continue purchasing the vaccine; they may ask citizens to pay for it. The international community should rethink the approach to ensure the sustainability of initiatives to ensure free access to the COVID-19 vaccine in the global south.

That notwithstanding, indirect costs hinder COVID-19 vaccine uptake in most African nations, such as transportation expenses to the health facility and loss of productive hours during the vaccination wait time.^73^ The governments of African nations should discuss these salient indirect costs and how to alleviate them, because resolution of these issues may improve COVID-19 vaccine uptake, especially among vulnerable groups, which includes older adults residing in the rural areas. We suggest that governments transport the vaccine to the rural communities if possible, or collaborate with nongovernmental organizations to subsidize or provide free transportation to vaccination centers. Several policies and strategies, including the “reaching every district approach”^100^ and the “door-to-door massive vaccination campaign,”^101^ have proved to increase vaccination in some African nations. The governments of African nations can consider whether these approaches could increase COVID-19 vaccine uptake because the structures exist in most health-care services.

Consistent with the previous review,^3^ activation, as defined earlier, is the extent to which individuals are nudged toward vaccine uptake, and is rarely studied in the literature. We found only one study that reported that participants were nudged toward using protective measures against the COVID-19 virus rather than acquiring the vaccine.^79^ Typical examples of nudges describing choice options are warnings/graphics, reminders, precommitment, and feedback.^102^ The use of pictures (as a warning) or reminders to depict the benefits of getting the COVID-19 vaccine, and the implications of not getting the vaccine could improve uptake. For instance, to increase social connectedness during the nationwide lockdown, we (the Emerging Researchers & Professionals in Ageing–African Network; https://erpaan.org/) created a visible paper reminder called “Connect with Parents Reminder Signage” placed at various clinics.^103^ This reminder helped clinicians prompt their patients to connect with their parents and grandparents in Nigeria socially through telephone.^103^ This approach is a typical example of a reminder as a nudge. The governments of African nations can apply similar concepts to increase COVID-19 vaccine uptake among older adults in Africa. For instance, nationwide signage called “Remind Your Parents and Grandparent to Get the COVID-19 Vaccine” should be launched in strategic areas in the cities. This approach is promising, because it was successful when we applied a similar approach during the nationwide lockdown in Nigeria.^103^ This simple and cost-effective approach to nudging individuals to get the COVID-19 vaccine can be implemented easily across various health clinics, because individuals will more likely get the vaccine when recommended by their health-care professionals.^32^

Although this review mapped out the literature concerning the 5As of COVID-19 vaccination, there are limitations. Because some of the national journals in Africa are not indexed in MEDLINE or related databases,^104^ we may have missed some studies. Furthermore, including articles published in English and French only may have led to missing some articles/reports. The mapping review only provides a surface-level description and lacks a critical and robust analysis of concepts explored.^15^ However, our study highlighted how concepts, including acceptability, accessibility, affordability, awareness, and activation relating to COVID-19 vaccine uptake, are explored in the African context. Because of the ambiguity and possible overlap of the 5As, we may have mis-mapped some articles. As suggested by Thomas et al.,^3^ the use of the 5A is for a practical purpose: to facilitate classification and easy identification of factors influencing vaccine uptake. Therefore, readers should reflect on this when interpreting the findings of this review.

Currently, no published evidence explores acceptability, acceptability, accessibility, affordability, awareness, and activation of COVID-19 vaccine uptake among the older adult (≥ 55 years) population in Africa. Although 68 articles explored these concepts across adults (≥ 18 years ) in Africa, acceptability was studied primarily, with activation rarely studied. We grouped factors affecting acceptability into sociodemographic factors; knowledge-, attitude-, and belief-related factors; COVID-19 vaccine efficacy and safety concerns; and trust in government and public health authorities.

## Supplemental Material


Supplemental materials
