# Supplementary material for: A Mapping Review on the Uptake of the COVID-19 Vaccine among Adults in Africa Using the 5As Vaccine Taxonomy
Source: Am J Trop Med Hyg. 2022 May 9;106(6):1688–97. doi: 10.4269/ajtmh.21-0515 (PMC9209920; doi:10.4269/ajtmh.21-0515)
Supplement: Supplementary file 1 [file tpmd210515.SD1.pdf]

Supplemental Table 1: included studies characteristics and findings related to 5As' concepts studied

Authors name, Year,<br><br>Country	Study design<br><br>Sample size<br><br>Mean Age	Articles findings per concepts studied				
		**Acceptance**	**Access**	**Affordability**	**Awareness**	**Activation**
Abebe et al., 2021<br><br>Ethiopia	Cross-sectional survey study<br><br>492<br><br>27.3	62.6% will accept COVID-19.<br><br>44.7% have a positive attitude toward the COVID-19	X	X	74% have good knowledge	X
Acheampong et al., 2021<br><br>Ghana	Cross-sectional survey study<br><br>2345<br><br>32.2	Almost a fifth (21%) of the respondents were unlikely to take the vaccine, while another 28% were undecided.	X	X	Information about herd immunity threshold in Ghana was reported possible if the preventive vaccination programs are combined with an enhanced and coordinated public education campaign	X
Adebisi et al., 2021<br><br>Nigeria	Cross-sectional survey study<br><br>517<br><br>35.7	Most of the respondents, 385 (74.5%) intend to take the COVID-19 vaccine when it becomes available.	X	X	X	X
Adeniyi et al., 2021<br><br>South Africa	Cross-sectional survey study<br><br>1308<br><br>(Not reported)	90% of the participants will accept COVID-19 Vaccine<br><br>Positive attitudes toward the SARS-CoV-2 vaccine were all significantly associated with vaccine acceptance	X	X	X	X

Africa CDC, 2021<br><br>15 countries: Burkina faso, Cote d;Ivoire, DR Congo, Ethiopia, Gabon, Kenya, Malawi, Morocco, Niger, Nigeria, Senegal, South Africa, Sudan, Tunisia, Uganda	Cross-sectional survey study<br><br>15699<br><br>41.5	Almost 1 in 5 of the participants said they would take the vaccine, even if it is not safe and effective	Access to Vaccine is limited across the regions of Ethiopia	X	X	X
Africa CDC, 2020<br><br>Africa (but not specific which country)	Cross-sectional survey study<br><br>1500<br><br>41.6	Over half of the participants 55% would take vaccine when accessible to them	Access to vaccine is a major concern to residents of most African countries as most rural communities are excluded	X	Public enlightenment should be strengthened across Africa	X
Agyekum et al., 2021<br><br>Ghana	Cross-sectional survey study<br><br>234<br><br>36.7	Concerns about the safety of vaccines (n = 93, 65.5%) and the adverse side effects of the vaccines (n = 23, 14.8%) were identified as the main reasons why health care workers would decline uptake of COVID-19 vaccines in Ghana.	X	X	X	X
Ahmed et al., 2021<br><br>Somalia	Cross-sectional survey study<br><br>4543<br><br>23.5	COVID-19 vaccine acceptance rates among students were similar to acceptance rates of other survey participants, suggesting that similar acceptance rates could be expected in a less biased Somalian study population.	X	X	X	X
Alice et al., 2021<br><br>Nigeria	Cross-sectional survey study<br><br>1228<br><br>32.8	Older age, male gender, trust in government, trust in public health authorities, confidence in vaccine developers, willingness to pay for and travel for a vaccine, and vaccination during an outbreak were significantly associated with COVID-19 vaccine acceptance.	X	X	X	X

Alhassan et al., 2021<br><br>Ghana	Cross-sectional study<br><br>1605<br><br>31.9	70% will accept the COVID-19 vaccine<br><br>Female HCWs (AME = − 11, SE = 0.04, $p < 0.05$) and those with lower educational qualification were less likely to accept a COVID-19 vaccine (AME = − 0.16, SE = 0.08, $p < 0.1$).	X	X	X	X
Arce et al., 2021<br><br>6 countries: Burkina Faso, Mozambique, Nigeria, Rwanda, Burundi Sierra Leone, Uganda	Cross-sectional survey study<br><br>44,260<br><br>38.4	Acceptance to take a COVID-19 vaccine across LMIC samples, ranging from 67% (Burkina Faso) to 97% (Nepal). Acceptance was considerably higher in LMICs (80%) than in the United States (65%) and Russia (30%).<br>Concern about side effects (40%) was the most common reason for reluctance. Health workers were considered the most trusted sources	X	Health workers were considered the most trusted sources	X	X
Arce et al., 2021<br><br>7 countries: Burkina Faso, Mozambique, Rwanda, Sierra Leone, Nigeria, and Uganda	Cross-sectional survey study<br><br>Not reported<br><br>Not reported	Vaccine acceptance in LMICs is primarily explained by an interest in personal protection against COVID-19, side effects is the most common reason for hesitancy.	X	X	Public health workers should be involved in awareness about COVID-19 vaccine	X
Belsti et al., 2021<br><br>Ethiopia	Cross-sectional survey study<br><br>425<br><br>38.6	The study found that only 31.4% were willing to take the COVID-19 vaccine. Being female, older age, marital status, rural residence, occupations, not having a health-related job, religion, educational status were statistically significantly associated with willingness to receive the COVID-19 vaccine.	X	X	There is need for increased awareness on the uptake of COVID-19 Vaccine	X
Bono et al., 2021<br><br>5 countries: Democratic Republic of Congo, Benin, Uganda, Malawi, and Mali	Cross-sectional survey study<br><br>10,183<br><br>45.1	The prevalence of vaccine acceptance increased from 76.4% (90% effectiveness) to 88.8% (95% effectiveness). Considering a 90% effective vaccine, Malaysia, Thailand, Bangladesh, and five African countries (Democratic Republic of Congo, Benin, Uganda, Malawi, and Mali) had lower acceptance odds compared to Brazil.	X	X	X	X

		Individuals who perceived taking the vaccine as important to protect themselves had the highest acceptance odds (aOR 2.49) at 95% effectiveness. Vaccine acceptance was also positively associated with COVID-19 knowledge, worry/fear regarding COVID-19, higher income, younger age, and testing negative for COVID-19.				
Botwe et al., 2021<br><br>Ghana	Cross-sectional study<br><br>108	59.3% were willing to have the vaccine.<br><br>Doubts about the vaccine's efficacy and side effects, conspiracy theory concerns about its effects on the Ghanaian race and fertility concerns were some reasons for their hesitance to receive the vaccine	X	X	X	X
Carcelen et al., 2021<br><br>Zambia	Cross-sectional survey study<br><br>2, 400<br><br>42.4	There was high acceptability of COVID-19 vaccination of their children, but substantial uncertainty and hesitancy about receiving the vaccine themselves.	X	X	X	X
Chiedozie et al., 2021<br><br>Nigeria	Cross-sectional survey study<br><br>499<br><br>35.1	51.1% were willing to take a COVID-19 vaccine, 30.5% were not willing as 18.4% were indecisive.	X	X	98% are aware of the vaccine	X
Dereje et al., 2021<br><br>Ethiopia	Mixed method<br>422<br><br>34.1	One out five (19.1%) participants were not willing to get vaccinated when it becomes available.	X	X	Nearly half (46.7%) of the participants exhibited poor level of knowledge.	X
Dinga et al., 2021<br><br>Cameroon	Mixed method<br><br>2512<br><br>34.3	15.4% of Cameroonians adults will accept the COVID-19 vaccine.	X	X	X	X

Ditekemena et al., 2021<br><br>Congo	Cross-sectional survey study<br><br>4131<br><br>40.6	Overall, 2310 (55.9%) indicated they were willing to be vaccinated.	X	X	X	X
Dula et al., 2021<br><br>Mozambique	Cross-sectional survey study<br><br>18778<br><br>38.5	Overall vaccine acceptability was 71.4% (86.6% among healthcare workers, 64.8% among other respondents; p < 0.001).	X	X	X	X
Eniade et al., 2021<br><br>Nigeria	Cross-sectional survey study<br><br>368<br><br>29.4	Two-fifth (40.5%) of respondent reported their willingness to take the COVID-19 if made available. Majority (69.8%) of those that are willing to take the vaccine would prefer a live attenuated form and 39.6% would prefer the vaccine administered intramuscularly.	X	X	X	X
Echoru et al., 2021<br><br>Uganda	Cross-sectional survey study<br><br>1,067<br><br>36.1	The acceptance rate for COVID-19 vaccination was (53.6%; 572/1067) and was also associated with those aged 18–20 years, males, elites at tertiary level of education (degree or diploma), students, Muslims, married, non-salary earners and rural dwellers having better odds and likeliness to accept vaccination.	X	X	X	X
Faezi et al., 2021<br><br>11 African countries: South Africa, Morroco, Ethopia, Democratic republic of congo, Cameroun, Mauritania, Mali, Burkinafaso, Cote d'ivoire, Guinea, Senegal and other	Cross-sectional survey study<br><br>2345<br><br>45.7	Overall, 66.81% of the contributors would like to be vaccinated against COVID-19, while %33.19 did not intend to be vaccinated.	X	X	Reasons for COVID-19 vaccine acceptance were related to awareness about vaccine side effects, fear of getting sick from the uptake of the vaccine, and getting accurate vaccine promotion news .	X

countries: USA, UK, European, Russia, China, South Korea, India, Iran						
Guangul et al., 2021<br><br>Ethiopia	Cross-sectional study<br><br>668<br><br>Not reported	The vaccine acceptance rate was 72.2%<br><br>Among the fears/concerns were vaccine adverse reaction, effectiveness of the vaccine, vaccine was made too fast to be safe, and COVID-19 from the vaccine.	X	X	X	X
Hoque et al., 2020<br><br>South Africa	Cross-sectional survey study<br><br>346<br><br>35.1	The study found a 63.3% acceptance of a COVID-19 vaccine, but there were noticeable demographic, knowledge, attitude and practice disparities observed in vaccine acceptance among pregnant women.	X	X	X	X
Ilesanmi et al., (2021)<br><br>Nigeria	Cross-sectional<br><br>440<br><br>37.22	63.0% strongly agreed to take the COVID-19 vaccine when produced	71.6% strongly agreed that COVID-19 get administered to everyone.	18.4% of the respondents were willing to pay for the prospective COVID-19 vaccine; 45 (55.6%) were willing to pay at least 5000 naira ($13.16).	67.30% of the respondents had heard of the prospective COVID-19 vaccine.	X
Iliyasu et al., 2021<br><br>Nigeria	Mixed method study<br><br>248<br><br>37.9	24.3% of the participants were willing to accept COVID-19 vaccine.<br><br>Acceptance was low among females, nurse/midwives, persons not tested for COVID-19, and those who perceived themselves as not at risk.	X	X	X	X
IPSOS, 2020<br><br>South Africa and 14 other countries (Canada, the	Cross-sectional survey study	Globally, 52% say they would become vaccinated within three months after the COVID-19 becomes available to all.	Only one-third of the population in the 15 countries have access to covid vaccines	X	X	X

United States, Australia, Brazil, China (mainland), France, Germany, Italy, Japan, Mexico, Russia, South Korea, Spain, and the United Kingdom)	20,000<br><br>45.1					
IPSOS, 2020<br><br>South Africa and 14 other countries (Canada the United States, Australia, Brazil, China (mainland), France, Germany, Italy, Japan, Mexico, Russia, South Korea, Spain, and the United Kingdom)	Cross-sectional survey study<br><br>13,500<br><br>45.6	64% said that they will get vaccine if they were available, below the global average (74%) of people would accept the covid-19 vaccine. The most common reasons given by those who indicated they would not get a vaccine was worry about side effect (53%), followed by doubt abouts its effectiveness (24%), followed by the perception of not being enough at risk from COVID-19 (16%). Almost a quarter (23%) of SA who do not intend to take the vaccines when available, indicated that they are opposed to vaccines in general.	X	X	X	X
Kanyanda et al., (2021)<br><br>6 countries (Burkina Faso, Ethiopia, Malawi, Mali, Nigeria and Uganda)	Cross-sectional country comparable, descriptive study based on a longitudinal survey<br><br>21,471<br><br>36.1	Acceptance rates in the six sub-Saharan African countries studied are generally high, with at least four in five people willing to be vaccinated in all but one country. Vaccine acceptance ranges from nearly universal in Ethiopia (97.9%, 95% CI 97.2% to 98.6%) to below what would likely be required for herd immunity in Mali (64.5%, 95% CI 61.3% to 67.8%).	X	X	X	X
Kanyike et al., 2021<br><br>Uganda	Cross-sectional survey study<br><br>600	COVID-19 vaccine acceptability was 37.3% and vaccine hesitancy 30.7%. Factors associated with vaccine acceptability were being male (adjusted odds ratio (aOR) = 1.9, 95% CI 1.3–2.9, p=0.001) and being single (aOR= 2.1, 95% CI 1.1–3.9, p=0.022). Very high	X	X	Awareness about COVID vaccine should also be extended to other students in the schools	X

	24.1	(aOR= 3.5, 95% CI 1.7–6.9, p<0.001) or moderate (aOR =2.2, 95% CI 1.2–4.1, p=0.008) perceived risk of getting COVID-19 in the future, receiving any vaccine in the past 5 years (aOR= 1.6, 95% CI 1.1–2.5, p=0.017), and COVID-19 vaccine hesitancy (aOR 0.6, 95% CI 0.4–0.9, p=0.036).				
Khairy et al., 2021<br><br>Sudan	Cross-sectional study (preprint)<br><br>576<br><br>35	57% of the participants were willing to get the vaccine.<br><br>Males were twice to four times more likely to accept the vaccine.	X	X	X	X
Khalis et al., 2021<br><br>Morrocco	Cross-sectional study<br><br>303<br><br>NR	62% Healthcare workers would accept vaccine<br><br>Participants were more likely to accept the COVID-19 vaccine if they were physician, nurse, or technician (OR 1.79; 95% CI: 1.09–2.95), had high score of confidence in the information circulating about COVID-19 (OR 1.91; 95% CI: 1.36–2.69), or had high score of perceived severity of COVID-19 (OR 1.55; 95% CI: 1.11–2.15).	X	X	X	
Kollamparambil et al., 2021<br><br>South Africa	Cross-sectional study	Vaccine acceptance is estimated at 70.8%<br><br>Higher awareness of COVID19 related information and higher household income are correlated with lower vaccine hesitancy. The non-black African population group has significantly high vaccine hesitancy compared to black Africans.	X	X	X	X

Lazarus et al., 2021<br><br>South Africa &Nigeria	Cross-sectional survey study<br><br>13,426<br><br>37.4	Age, gender, and education were not associated with COVID-19 vaccine acceptance in a Nigerian population. However, age was significantly associated with COVID-19 Vaccine acceptance (if recommended by employer) in the SA population, while gender and education were not.	X	X	X	X
Lazarus et al., 2021<br><br>South Africa and Nigeria	Cross-sectional online surveys<br><br>13,426<br><br>46.6	81.85% and 58.89% of participants in SA and Nigeria are willing to take COVID-19 when available.	X	X	X	X
Mannan & Farhana 2020<br><br>10 countries: Algeria, Egypt, Botswana, Kenya, Libya, Mali, Mauritius, Morocco, Nigeria and South Africa	Cross-sectional survey study<br><br>26, 852<br><br>36.2	Acceptance of COVID-19 were 66.32% in Algeria, 43.44% in Egypt, 71.23% in Bostwana, 61.33% in Kenya, 49.63% in Libya, 62.44% in Mali, 82.75%in Mauritius, 48.44% in Morocco, 61.54% in Nigeria and 79.26% in South Africa	X	X	X	X
McAbee et al., 2021<br><br>Zimbabwe	Cross-sectional survey study<br><br>551<br><br>41.3	More than half (55.7%) of the respondents reported intending to vaccinate themselves or their households. Multivariate logistic regression indicated that the likelihood of vaccine intentions was most strongly associated with confidence in vaccine safety.	X	X	X	X
Mesele, 2021<br><br>Ethiopia	Cross-sectional study<br><br>415<br><br>Not reported	45.5% of the participants will accept the vaccine.<br><br>Sex, education, mass media, received any vaccination during childhood, family members or friends being diagnosed with COVID-19 and those tested positive	X	X	X	X

		for COVID were significantly associated with acceptance				
Mesfin et al., 2021<br><br>Ethiopia	Cross-sectional<br><br>398<br><br>32.2	The intention to take COVID-19 vaccine among HIV-positive patients was 33.7%.<br><br>The probability of PLWHA to take COVID-19 vaccine was two (AOR = 2 (1.08–3.44)) times higher among those who have been diagnosed with chronic diseases (exclude HIV) with those with not been diagnosed. PLWHA who were male in sex were five (AOR = 5 (2.96–8.68)) times more likely to take COVID-19 vaccine than female. The study also indicated that the odds of intention to take the COVID-19 vaccine were 4.1 times (AOR = 4.1 (2.33–7.31)) times higher among those participants who had good knowledge of COVID-19 practice compared with those who had poor knowledge.	X	X	X	X
Mose & Yeshaneh 2021<br><br>Ethiopia	Cross-sectional survey study<br><br>396<br><br>34.1	COVID-19 vaccine acceptance was found to be 70.9%. Maternal age (34–41) years, primary maternal educational status, good knowledge, and good practice of pregnant women towards COVID-19 and its preventive measures were factors associated with COVID-19 vaccine acceptance.	X	X	X	X
Mohamed et al., 2021<br><br>Egypt	Cross-sectional (preprint)<br><br>496<br><br>32.1	Only 13.5% totally agree to receive the vaccine, 32.4% somewhat agree and 40.9% disagreed to take the vaccine.<br>.	X	X	X	X
Narnia et al., 2021<br><br>South Africa	Cross-sectional survey study (Round 3 of the UJ-HSRC	2/3 of the participants favours vaccines. 67% said yes, they will take the vaccine when it is available to them, 52% and 14% said they will definitely or probably get the vaccine.	X	X	X	X

	Covid-19 Democracy<br><br>10, 618<br><br>32.1					
Ngasa et al., 2021<br><br>Cameroon	Cross-sectional study (preprint)<br><br>371<br><br>29.1	45.38% indicated willingness to accept the vaccine if offered. The most common reason advanced for non-acceptance of the vaccine was negative perceptions about the efficacy of the vaccines.	X	X	X	X
Ngoyi et al., 2020<br><br>Democratic Republic of Congo	Cross-sectional survey study<br><br>438<br><br>26.5	Only 25.3% of students said they would receive the covid-19 vaccine if it was available. After logistic regression, male students (AOR = 2.24 [1.40-3.58]; p = 0.001), those who regularly consult official sites (AOR = 0.24 [0.90-0, 68]; p = 0.004) and students with poor knowledge of Covid-19 (AOR = 1.73 [0.45-0.02]; p =0.000) were significantly associated with the desire to be vaccinated.	X	X	X	X
Nzaji et al., 2020<br><br>Congo	Cross-sectional survey study<br><br>613<br><br>40.3	Only 27.7% of HCWs said that they would accept a COVID-19 vaccine if it was available.<br><br>Having a positive attitude towards a COVID-19 vaccine (OR =11.49; 95% CI: 5.88–22.46) was significantly associated with reporting willingness to be vaccinated.	X	X	Knowledge of COVID-19 was not associated with willingness to received COVID-19	X
Olomofe et al., 2021<br><br>Nigeria	Cross-sectional survey study (Preprint)<br><br>776<br><br>38.1	58.2% participants were willing to take a potential COVID-19 Vaccine. For vaccine uptake, being male (p= 0.002) and the perception that “vaccines are good” (p< 0.001) were the positive predictor of uptake of a potential COVID-19 vaccine.	X	X	X	X

Oyekale, 2021<br><br>Ethiopia	Cross-sectional survey study<br><br>2,178<br><br>33.1	Majority of the respondents (92.33%) would receiveCOVID-19 vaccines.	X	X	X	X
Runciman et al., 2021<br><br>South Africa	Cross-sectional survey study with embedded qualitative open-ended questions<br><br>10, 618	67% said they would definitely or probably take a vaccine; 18% said they would definitely not or probably not take the vaccine and 15% were unsure if they would take the vaccine. The explanations for vaccine acceptance include protect oneself (29%) and protect other (25%), For those who were either unsure or against taking the vaccine the most common explanations related to concerns about side-effects (25%) and concerns about the overall effectiveness of the vaccine (18%). Explanations related to conspiracy theories, or the occult did not appear frequently, 7% and 4% respectively.	X	X	X	X
Saied et al 2021<br><br>Egypt	Cross-sectional survey study<br><br>2,133<br><br>20.2	71% of the students have the intention to receive COVID-19 but may postponed, 13% said they will receive immediately it is available	X	X	X	X
Shehata et al., 2021<br><br>Egypt	Cross-sectional study<br><br>1268<br><br>Not reported	24% express their acceptance towards COVID-19 vaccine,<br><br>Age, gender, higher educational attainments, prior infection, lack of direct patients' contact, and working in rural health facilities were determinants of vaccine acceptance	X	X	X	X
Terefa et al., 2021<br><br>Ethiopia	Cross-sectional study	324 (62.1%) of them were vaccinated with any of the COVID-19 vaccines at least once.	X	X	X	X

	522<br><br>Not reported	The study indicated that COVID-19 vaccine uptake was associated with age range from 35 to 44 years [AOR = 12.97, 95% CI: 2.36– 71.21], age beyond 45 years [AOR = 18.95, 95% CI = 2.04– 36.29], being male [AOR = 2.91, 95% CI = 1.05,8.09], being only an academician [AOR = 0.23, 95% CI: 0.10– 0.49], academicians working in University hospitals [AOR = 0.19, 95% CI: 0.05– 0.83], perceiving their family as healthy [AOR = 4.40, 95% CI: 2.21–8.75], no history of receiving other vaccine before as an adult [AOR = 4.07, 95% CI: 2.07– 8.01] and no history of contact with confirmed COVID-19 patients or clients [AOR = 0.42, 95% CI: 0.20– 0.86].				
Zewude & Habtegiorgis, 2021<br><br>Ethiopia	Cross-sectional survey study<br><br>491<br><br>32.1	Research participants generally had a low (46.1%) willingness to take a COVID-19 vaccine. The main reason for most (37%) respondents' hesitancy to take the vaccine is found to be the concern over the safety and/or the side effects of the vaccine (37%), followed by doubt about the vaccine's effectiveness (20.7%), and lack of adequate information (12.7%).	X	X	X	Participants are nudge towards using protective measures rather than vaccine
Opinion papers commentary and organisational reports						
Acharya et al., 2021<br><br>(Low- and Middle-income African countries)	Opinion paper	Low SES affects vaccine acceptance among Africans	People from urban slums and marginalized and migratory populations have poor access to immunization facilities.	Low SES affects vaccine purchase	X	X
Afolabi & Ilesanmi, 2021	Opinion paper	Multisectoral collaboration will enhance acceptance COVID-19 vaccine across African countries	COVAX provision of the COVID-19 vaccine is a viable strategy to ensure a timely availability of	For low-income countries, who are not self-sufficient to purchase the	Information around COVID-19 vaccine awareness is important in addressing	X

Nigeria			the COVID-19 vaccine for developed countries	COVID-19 vaccine, COVAX provides a lifeline	misconceptions	
Bangalee & Suleman 2020<br><br>South Africa	Opinion paper	X	COVID-19 has created an opportunity for international cooperation in working on transparency issues to ensure equitable sharing of COVID-19 vaccine across African countries specially in South African country. limited supply or sole proprietary rights to sell the vaccine introduces the threat of vaccine price gouging.	X	X	X
Choi, 2021<br><br>Africa, China, Russia and India	Opinion paper	X	There is a patchwork of different pathways for countries to access newly developed COVID-19 vaccines, including direct purchase, group financing and donations.	The African Export-Import Bank is offering loans to individual Afrian Union member states to finance the COVID-19 vaccinantion immunisation programme.	X	X
Ekwebelem et al., 2021<br><br>16 countries: Burkina Faso, Cote d'Ivoire, Democratic Republic of the	Opinion paper	Acceptance of COVID-19 Vaccine is by will and voluntary.	X	X	X	X

Congo, Ethiopia, Gabon, Kenya, Malawi, Morocco, Niger, Nigeria, Senegal, South Africa, Sudan, Tunisia, and Uganda						
Figuero & Bottazzi, 2021<br><br>Lancet Commission on COVID-19 Vaccines and Therapeutics Task Force Members	Commentary	X	The COVAX Facility of the ACT Accelerator has agreements to access 2 billion doses of WHO pre-qualified vaccines during 2021, but this represents only 20% of the vaccine needs of participating countries.	X	X	X
IPSOS, 2020<br><br>Africa (not specific)	Opinion Paper	X	There were problems with vaccine access in most of the countries in the African region	X	X	X
Jerving, 2021<br><br>Africa (not specific)	Opinion Paper	X	There are concerns that COVAX's access to vaccines could again be compromised by disruptions in the global supply chain`		There are limited level of awareness of covid vaccine in the rural African region	
Loembé & Nkenganson, 2021	Opinion paper	X	Vaccine access in Africa today is a case of history repeating itself, with the infamous episodes of inequitable access to HIV therapies or hoarding of the	X	X	X

Africa (not specific)			H1N1 vaccine doses by a few during the 2009 outbreak being re-enacted			
Machingaidze, & Wysonge, 2021<br><br>Africa		Promoting the uptake of vaccines (particularly those against COVID-19) will require understanding whether people are willing to be vaccinated, the reasons why they are willing or unwilling to do so, and the most trusted sources of information in their decision-making.	Access to smartphones in LMIC was great tool for self-education, which is key to vaccination decision-making. Although it also posse several challenges in form of misinformation.	X	X	X
Makoni 2021<br><br>Tanzania	Commentary	X	Government of Tanzania refused to provide COVID-19 Vaccine inspite of international aids and support for the country. Tanzania stopped releasing official COVID-19 statistics	X	X	X
Nkengasong, et al, 2020<br><br>Africa (not specific)	Commentary	X	A 'whole of Africa' coordinated approach is needed to prepare for access and roll-out of a COVID-19 vaccine	A 'whole of Africa' coordinated approach is needed to prepare for the development, purchase of COVID-19 vaccine	X	X
Samarasekera, 2021<br><br>South Africa	Commentary	X	Although concerns over equitable access to the vaccines and country preparedness continue, another key issue for the continent is vaccine	X	X	X

			confidence.			
Wlysonge et al, 2021<br><br>Ethiopia	Opinion paper	Research should investigate how people think, feel, and act in relation to a vaccine when developing strategies to generate acceptance and uptake for the vaccine.	Unwillingness to receive vaccines when vaccination services are available and accessible is significant problem in most developing countries	X	X	X

Note: X indicates that the study did not report on the concepts.
Pre-print is an article that have been submitted for peer review, but has not been reviewed, but deposited in a preprint server for access.
